# Replacement of refined sugar by natural sweeteners: focus on potential health benefits

**DOI:** 10.1016/j.heliyon.2022.e10711

**Published:** 2022-09-20

**Authors:** Shiza Arshad, Tahniat Rehman, Summaya Saif, Muhammad Shahid Riaz Rajoka, Muhammad Modassar Ali Nawaz Ranjha, Abdo Hassoun, Janna Cropotova, Monica Trif, Aqsa Younas, Rana Muhammad Aadil

**Affiliations:** a National Institute of Food Science and Technology, University of Agriculture, Faisalabad 38000, Pakistan; b Food and Feed Immunology Group, Laboratory of Animal Food Function, Graduate School of Agricultural Science, Tohoku University, Sendai 980-8572, Japan; c Institute of Food Science and Nutrition, University of Sargodha, 40100 Sargodha, Pakistan; d Univ. Littoral Côte d’Opale, UMRt 1158 BioEcoAgro, USC ANSES, INRAe, Univ. Artois, Univ. Lille, Univ. Picardie Jules Verne, Univ. Liège, Junia, F-62200, Boulogne-sur-Mer, France; e Sustainable AgriFoodtech Innovation & Research (SAFIR), 62000 Arras, France; f Department of Biological Sciences Ålesund, Faculty of Natural Sciences, Norwegian University of Science and Technology, Larsgårdsvegen 4, 6025 Ålesund, Norway; g Department of Food Research, Centre for Innovative Process Engineering (Centiv) GmbH, 28857 Syke, Germany

**Keywords:** Refined sugar replacement, Natural sweetener, Health benefits

## Abstract

Refined sugar is a processed product containing 99% sucrose, which is obtained from sugarcane (70%) or sugar beet (30%). In modern societies, sugar continues to play a significant role in the diet, recognised not only for its flavour and special sweetening properties but also for its role in food preservation. On the other hand, a high consumption of refined sugar is associated with non-communicable diseases and many health issues such as a high risk of dental caries, overweight and neurodevelopmental disorders in children. Alternatives like unrefined sugars have generated a lot of interest as a healthy substitute due to their nutraceutical properties. This paper is aimed to review the beneficial effects of sugar derived from natural sources and highlight health problems that could be caused by refined processed sugar. Refined sugar is frequently used in variety of items including processed foods, soft drinks or ice creams although it is considered unhealthy due to its high salt and sugar content as well as added fats and artificial coloring. Natural sugars are preferred because they have a high nutritional value and a high concentration of healthy compounds, which offset the negative effects of refined sugar. Therefore, removing refined sugar or at least reducing its consumption should be promoted as a healthier option in food choices.

## Highlights

• Beneficial effects of natural sources of sugar over refined sugar.
• Health problems that could be caused by refined/processed sugar.
• Reduction of refined sugar and thus replacing it with better and nutritional alternatives.

## 1 Introduction

Sugar has become an inseparable part of many food cultures. Besides being an imperative element in the production of various sweets, sugar has many beneficial properties in foods such as preservatives, bulking agents, texturizers, moisturizers, dispersants, stabilizers, fermentation substrates, flavor carriers, and browning and decorative agents (Manickvasagan et al., 2017). The term “sugars” refers to mono- and di-saccharides in terms of chemical categorization. Glucose, fructose, and galactose are the three main monosaccharides – hexoses (six-carbon sugars) that makeup naturally occurring di-, oligo-, and polysaccharides (Fidler Mis et al., 2017), while “Sugar” is referred to as sucrose also known as table sugar, which is made up from fructose and glucose units (Sachdev, 2018). Sugars that have an “exposed” carbonyl group are referred as reducing sugars whereas those that cannot “open” are referred as non-reducing sugars (sucrose) (Clemens et al., 2016).

Sugars are naturally present in foods like vegetables, fruits and milk but these are also added during preparation and processing or serving (Jomaa et al., 2021). Sugars naturally present in whole foods are an essential component of a healthy and well-balanced diet. Added sugars boost calorie intake, the sensory effect of meals, and overall satisfaction but they are not essential for good health and nutrition (Fidler Mis et al., 2017). They can substitute nutrient-dense foods and lead to poor health outcomes by delivering calories without other necessary elements such as vitamins, minerals, or other important nutrients (Slavin, 2012). According to the WHO, naturally occurring sugars, known as intrinsic sugars, are naturally incorporated within intact plant cell walls (Fidler Mis et al., 2017). In recent years strong concerns have been raised about excessive sugar intake and its effect on health. During the last fifty years, the consumption of sugar has tripled around the world. According to the global sugar glut, more than 500 calories per day on average from added sugar alone are eaten in different parts of the world (Lustig et al., 2012). This high calorie consumption is a result of using sugary foods and drinks that have been processed and made with caloric sweeteners, particularly table sugar, which is a form of refined sugar (Makarem et al., 2018). However, most reported data indicate that sugar intake is higher than the recommended level (Edwards et al., 2016). The WHO recommends reducing the intake of added sugar to <10% of the total energy (Organization, 2003). The U.S. Department of Agriculture's (USDA) food guide recommends consuming added sugar within the range of 6–10% of total energy (Manickvasagan et al., 2017).

Table sugar is widely referred to as one of the oldest sweetening agents and the most widely used caloric sweetener, both for home and industrial use. Sucrose is usually utilized in its refined and/or crystal form. This white refined sugar is produced from sugar beet or sugarcane industrially (Gerschenson et al., 2017). After extraction, purification, evaporation and crystallization of sugarcane juice, this sugar undergoes different refinement stages where it may lose some minerals. Then, additives such as clarifiers and preservatives are added, which remain in the products and decrease the nutritional quality. The purifying of white crystal sugar yields white refined sugar, which is characterized by grains that dissolve easily in drinks and other preparations. In order to make it whiter, chemical additions like sulphur are utilised during processing (Curi et al., 2017). In large-scale production and refining, chemicals including calcium hydroxide Ca(OH)_2_, lime (CaO), phosphoric acid, bleaching and de-coloring agents of varying concentrations and consistency are generally responsible for the impurity of white refined sugar (Sadjadi et al., 2018). Much like the classic drugs of abuse such as cocaine, alcohol, tobacco, and caffeine, refined, added sugars have similar impacts in terms of habit-forming. Foods with high sugar contents have been shown to produce drugs with psychoactive effects. The most accepted methods of sugar addition are sucrose and high-fructose corn syrup, which are widely used nowadays. Moreover, the extraction and refinement process of sugar into white crystal (refined sugar) is similar to cocaine from coca leaf (DiNicolantonio et al., 2018). The purpose of this review is to emphasize the need of reducing or eliminate the use of refined sugar in daily life, which is linked to a variety of health issues and focus on the potential health benefits that obtain from naturally present sugars.

## 2 Excess sugar – a source of diseases and disorders

**Figure 1:**
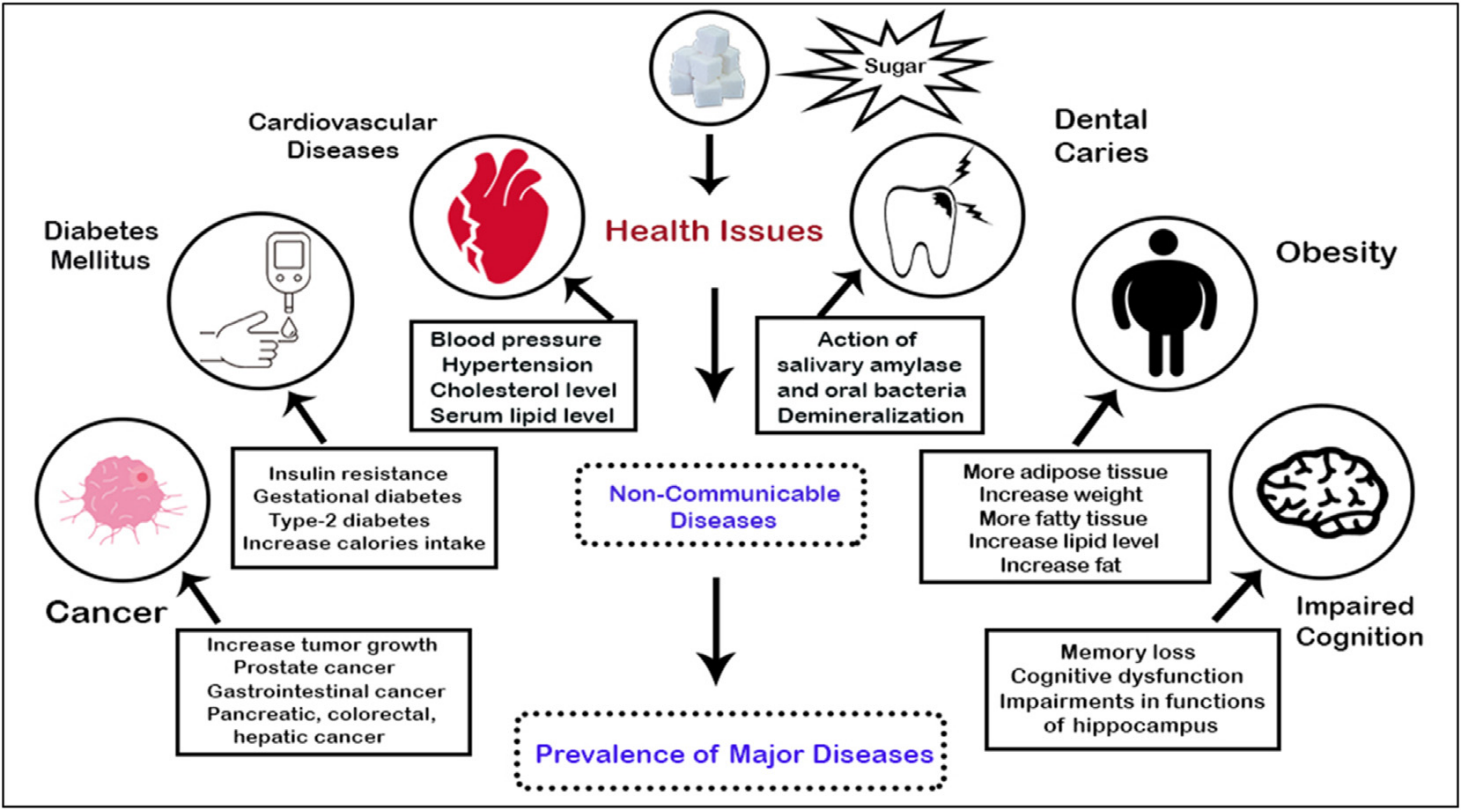
Mechanism of major health problems caused by refined sugar.

Sugar intake is a major public health problem that has gained popularity in recent years among people of all ages. Dietary sugar in excess raises the risk of metabolic conditions including obesity and diabetes as well as cardiovascular disorders (Alkhaldi et al., 2021). In many countries, different epidemiological studies and trials have associated a high consumption of sugar-sweetened beverages (one of the main dietary sugar sources) with weight gain, poor dental health, cancer, metabolic syndrome, heart disease and type 2 diabetes mellitus (Kaartinen et al., 2017). Mechanisms of some major health problems caused by consuming refined sugar are shown in Figure 1.

### 2.1 Oral health diseases

The Global Burden of Disease Study 2016 reported that half of the global population was affected by oral diseases. Any stage of life can experience oral illness, which can start as early as 18 months of age. A neglected area of global health is oral health, due to untreated dental decay 2.3 billion people are affected by permanent teeth and more than 560 million children are affected by primary teeth throughout the world (Fisher et al., 2018). The direct economic costs linked to the treatment of oral disease were estimated to be US$298 million/year in 2010, which accounts for 4.6% of the total expenditure on health globally (Listl et al., 2015). Oral health inequalities and improper oral health have economic and social effects on people and untreated oral diseases are the noticeable indicator of health inequalities in the community (Ravaghi et al., 2013). In the Sustainable Development Goal 3 (SDG 3), oral illness are considered comorbidity factors, including communicable and non-communicable diseases, sexual and reproductive well-being and maternal and infant health (Fisher et al., 2018). Furthermore, schoolwork and the learning process are also impaired by oral health problems. Children are likely to miss school because of untreated dental caries that cause oral pain or infection (Karki et al., 2019). Many recent systematic reviews were published reporting about those children having a high dental caries rate, low attendance and poor academic achievement in school (Rebelo et al., 2019; Ruff et al., 2019). For example, in Nepal, a cross-sectional analysis has shown that untreated dental caries and their effects have a significant impact on the quality of life associated with oral health that can lead to low school performance (Karki et al., 2019).

Globally, dental caries is a major public health problem, and it is the utmost predominant condition listed in the Global Burden of Disease Report 2015 placed first for permanent teeth decay. Dental caries is the most dominant non-communicable chronic disease, and it affects all age groups from children to older adults. Dental caries affects 80% of the world’s population and dietary sugar is the most substantial risk factor for dental caries (Moynihan, 2016). The caries procedure is an aftereffect of the intake of fermentable carbohydrates and sucrose (table sugar) and occurs in the mouth when bacteria process the sugars to produce some acids, which may demineralize the hard tissues of the teeth. People who have higher free sugar consumption have more dental caries (Hujoel and Lingström, 2017; Organization, 2017). During 19th century, it was found that restricting sugar prevents dental problems because most children were suffering from dental decay during that time. Sugar is known to increase the risk of gingival bleeding in addition to dental caries (Hujoel and Lingström, 2017). A study showed the effects of oral health optimized diet on gingival and periodontal inflammation. Participants of the experimental group had to change their diet including low carbohydrates, high rich in Omega-3 fatty acids, vitamin C, antioxidants and fiber for four weeks while control group participants did not do any changes in their dietary routine. The result showed that diet low in carbohydrate diet, high in Omega-3 fatty acids, vitamins C and rich in fibers can substantially reduce periodontal and gingival inflammation (Woelber et al., 2017).

A study showed that school nutrition policies, particularly those aiming to restrict sugar consumption, may have positive impacts on students' oral health, and are likely to minimize the healthcare costs linked with dental caries. An observational cross-sectional investigation was carried out to check the role of dietary sugar in the development of caries. Data were obtained from a questionnaire, 24-hour dietary recall interviews, and clinical examination of 128 subjects aged 11–12 years. Consumption of free sugars before bed and between meals was assessed through dietary assessment. A result of this study showed that a significant risk factor for dental caries may be consuming sweets before bedtime (Goodwin et al., 2017). Sugar intake is considered a vital risk for dental caries development, which is also likely to influence the etiology of rheumatic fever (Thornley et al., 2017).

### 2.2 Non-communicable diseases

Each year 56 million deaths occur worldwide, and non-communicable diseases (NCD) are responsible for 38 million (68%) deaths and 16 million of these deaths (more than 40%) were premature (before the age of 70). By 2030, the annual number of NCD deaths is expected to increase to 52 million. The growing pressure on NCDs raises difficulties for both households and the government to afford health care. This economic pressure also influences health and health-related behavior and reduces the quality of life (Jan et al., 2018). Sugar consumption is linked to an increase in NCD. The effects of sugar on the body can be similar to those of alcohol (Lustig et al., 2012). Increased sugar intake could increase the risk of obesity, diabetes mellitus, cardiovascular diseases, hypertension, cancer and intolerance to glucose (Manickavasagan et al., 2013). Foods with high sugars are the reason for empty calories with a minimal level of vital nutrients and dietary fibers. These foods replace nutrient-dense foods and contribute to overfed, and undernourished people at the same time (Steele et al., 2016). Many studies showed that excessive use of added sugar increases the risk of high cholesterol, blood pressure, hypertension, type 2 diabetes, obesity, and heart diseases as shown in Table 1.

**Table 1:** Studies show high sugar intake and its association with non-communicable diseases.

Study	Results	References
Drinking water was replaced with an 8% sucrose solution in rats and within a week tachycardia and hypertension developed	This animal study showed a strong link between the onset of arterial hypertension and the intake of highly refined sugar intake	(Goran et al., 2013)
From different countries, an online survey was conducted that included 2496 participants and questions about the consumption of soft drinks, the presence or absence of specific diseases, physical exercise and medication were asked.	A greater presence of obesity, gastritis constipation and mental illness among people who consume cola soft drinks more.	(Martín et al., 2018)
A cross-sectional study was carried out on 6–12 years of school-going children. Data about the consumption of sugar-containing foods and dental history were obtained.	Results of the human study indicate a high rate of dental caries and a rise with increased sugar consumption and this may be due to the increased availability of refined sugary products.	(Moynihan and Kelly, 2014)
Systematic review and meta-analysis of randomized controlled trials were examined that investigated effects on blood pressure and lipids from the alteration of dietary free sugars.	*A meta*-analysis Dietary sugars affect the serum lipids and blood pressure the partnership is independent of sugars' impact on body weight.	(Te Morenga et al., 2014)
In spontaneously hypertensive rats (SHR) the effects of dietary sucrose on various myocardial and hemodynamic parameters were studied following 6 week period. Rats consume a supplement of 10% sucrose in the drinking water.	Findings by reviewing scientific literature indicated that moderate dietary sucrose intake is consistent with cardiovascular changes that can further stress a heart already weakened by chronic hypertension.	(DiNicolantonio and O'Keefe, 2016)
Only a few prospective studies have examined the link between added sugar consumption and death from cardiovascular disease.	Many adults, In the US consume more added sugar than the one suggested for a healthy diet and found a significant association between added sugar intake and decreased risk of death from CVDs.	(Yang et al., 2014)
Six studies for type 2 diabetes mellitus, three for the metabolic syndrome, and one for coronary heart disease were included in a meta-analysis of ten prospective cohort studies.	Evaluated direct relation between consumption of sugar containing soft drinks and weight gain with cardio-metabolic disease risk.	(Bray, 2013)
*A meta*-analysis of observational studies was conducted, and data were obtained via articles published up to February 2013 on different websites	The result showed that intake of sugar-sweetened beverages (derived from caloric sweeteners like table sugar or sucrose, high fructose corn syrup) may raise the risk of Coronary heart diseases, particularly in males and American populations.	(Huang et al., 2014)

Epidemiologic evidence has constantly associated sugar and its products with higher incidence of weight gain, diabetes, and metabolic syndrome, which are considered as main factors for cancer development. Increased sugar intake is responsible for elevated blood glucose, which leads to elevated insulin-like growth factor 1, inflammation, obesity, and increased oxidative stress which causes cell proliferation and differentiation and leads to increased cancer risk (Makarem et al., 2018).

Refined sugars are considered the major foods that cause cancer, due to their role in the development of cancer cells Makarem et al. (2018). Systematically identified 37 prospective cohort studies reported that dietary sugars linked with the risk of cancer from PubMed, Embase, and CINAHL. Nearly 15 studies reported risk of cancer with total sugar, 14 for sucrose and fructose, 5 for added sugar, and 15 for sugary foods and drinks in 2–5 studies 60–95% and in 8–15 studies 23–200%, higher risk of cancer was observed with higher intake of sugar containing foods and drinks. These findings have linked dietary sugars to some lifestyle-related cancers including female cancers (breast, endometrial, and ovarian), prostate cancer, gastrointestinal cancers (pancreatic, colorectal, hepatic, and biliary tract), and hematologic (lymphoma, myeloma, and leukemia) cancers.

### 2.3 Biliary system disorder

Observational studies in humans have shown that higher refined sugar consumption is linked with higher gallstone prevalence. The correlation between refined sugar consumption and gallstones could be attributed to the fact that eating large amounts of sugar will contribute to obesity (Sharma and Tandon, 2012). A study proposed that the intake of processed carbohydrate foods (notably refined sugar) raised the capacity of bile cholesterol and causes formation of cholesterol gallstones. To check this hypothesis, 13 subjects (10 women and 3 men) suffering from cholesterol gallstones, consumed refined and unrefined carbohydrate diets for 6 weeks each. The lipid content of the duodenal bile and the kinetics of bile acid were determined after each diet and the results exhibited that the cholesterol concentration was higher in individuals who were on a processed carbohydrate diet. Bile cholesterol increases due to the consumption of carbohydrates in refined form. The risk of gallstones might be decreased by avoiding refined carbohydrate foods (Di Ciaula et al., 2019). Although it has not been proven that eating refined sugar encourages the development of gallstones, it would be advisable for people who are at risk of developing gallstones to prevent unnecessary refined sugar consumption (Sharma and Tandon, 2012).

### 2.4 Neurodevelopmental disorders in children

Attention Deficit Hyperactivity Disorder (ADHD) is a neurobiological disorder marked by persistent symptoms of hyperactivity, inattention, and impulsivity. ADHD starts in childhood and often lasts until adulthood, causing poor educational performance as well as lifelong accumulated losses, such as family and interpersonal difficulties. Many studies have shown that high consumption of processed sugar and saturated fat diets can elevate the risk of ADHD or hyperactivity (Del-Ponte et al., 2019). Howard et al. (2011) conducted a cohort study and concluded that ADHD symptoms were most common in adolescents that had a high score on a western diet that included a high amount of refined sugars and saturated fat. Peacock et al. (2011) and Abbasi et al. (2019) also showed that high sugar and fat diet increases the risk of ADHD.

It is perpetuated by the common belief that refined sugars induce hyperactivity. There are two hypotheses behind it; the first one is that after the consumption of sugar, certain children experience “true reactive hypoglycemia”, and the other hypothesis is that hyperactivity occurs due to an allergy to refined sugar. No recent inquiries have been carried out to determine the connection between sugar and behavior in children, although rat studies indicate that sugar dependency contributes to behavioral and neural changes which affect the dopamine system in a similar way when it is affected during stimulant sensitization (Wolraich et al., 2007).

### 2.5 Impairment in cognitive abilities

Refined sugars consumption is also linked with impaired cognitive functions. Although human research is lacking to investigate the long-term impact of sugar consumption on cognitive functions, it is understood that ingestion of sugar contributes to reduced insulin resistance and glucose tolerance, which in turn could be related to cognitive impairment. A diet high in saturated and refined sugar also associated with poorer memory performance and similar (Francis and Stevenson, 2013). It was found that the consumption of processed foods (processed meats, refined grains, high-fat dairy products, candies and chocolates) in a group of stroke-free adults (35–55 years) was correlated with the lower cognitive testing performance of vocabulary and verbal fluency as compared to those with a whole diet pattern (Akbaraly et al., 2009). Another study resulted that a high intake of sugar and saturated fat was connected with lesser performance on the neuropsychological memory task than that recorded a lower intake of sugar and fat. The results showed that the cognitive effects were perhaps due to the diet rather than weight gain or related disorders (Francis and Stevenson, 2011). High-sucrose diets can change cognitive behavior by reducing plasticity in the neurons. Many studies showed that high refined sugar and saturated fat diet could contribute to impaired performance of the Morris water maze test, which was correlated with reduced expression of neurotrophic hippocampal brain-derived factor, a major neural learning and memory mediator (Jurdak and Kanarek, 2009).

### 2.6 Complications during pregnancy

For optimal health of the mother and fetus, a nutritional diet is very important during pregnancy. Excess sugar consumption is the number one factor that can characterize an unhealthy diet because high consumptions of sugar products typically provide unnecessary calories and replace healthier foods containing essential macro and micronutrients. Consumption of sugar is linked with the high risk of preeclampsia, excess gestational weight gain and gestational diabetes mellitus in the mother. All of these factors may contribute to an greater chance of problems during pregnancy and perinatal mortality and morbidity (Graham et al., 2013). Excessive consumption of sugar during pregnancy causes weight gain not only in the mother but also in the offspring and contributes to the obesity epidemic (Jen et al., 2017). During pregnancy, consumption of sugar-containing beverages is positively correlated with the body mass index of children during early childhood and particularly with higher fat mass. Another research was conducted to investigate the effect of sugar-sweetened cola beverages during pregnancy and results showed that daily high consumption of sugar-sweetened cola beverages is linked with an increase in the rate of preterm delivery (Petherick et al., 2014).

In Denmark, a prospective cohort study of 46,262 women evaluated that excessive sugar intake was directly linked with gestational weight gain. The authors found that a higher protein to carbohydrate ratio was inversely associated with weight gain while consumption of excessive sugar was strongly associated with gestational weight gain. Increasing the protein-to-carbohydrate ratio with a focus on reducing the added sugar can help to reduce excessive gestational weight gain (Maslova et al., 2015). It was also seen that during pregnancy maternal sugar consumption is linked with an increased risk of atopic asthma and atopy in offspring, regardless of sugar consumption in early childhood (Bédard et al., 2017).

High sugar consumption during pregnancy is linked to the growth of gestational diabetes mellitus (GDM). A study based on National Health and Nutrition Examination Survey data investigated the impact of diet patterns on pregnant women and the risk of GDM. The results showed that a high-added sugar diet and low fruit and vegetable intake increased the risk for GDM (Shin et al., 2015). In another study, the relationship between pre-pregnancy sugar-sweetened soft drink (SSSD) consumption and risk for GDM was investigated (Donazar-Ezcurra et al., 2018). The study showed positive association between SSSD and GDM that persisted even after controlling potential confounders like a family history of diabetes, body mass index (BMI), total energy intake and fiber intake (Donazar-Ezcurra et al., 2018). Research on women's sugar consumption behavior during pregnancy concluded that strategies to help pregnant women to maintain a balanced diet and reduce the consumption of sugar should be driven by nutritional education and nutritional awareness (Graham et al., 2013).

## 3 Natural sources of sugar

**Figure 2:**
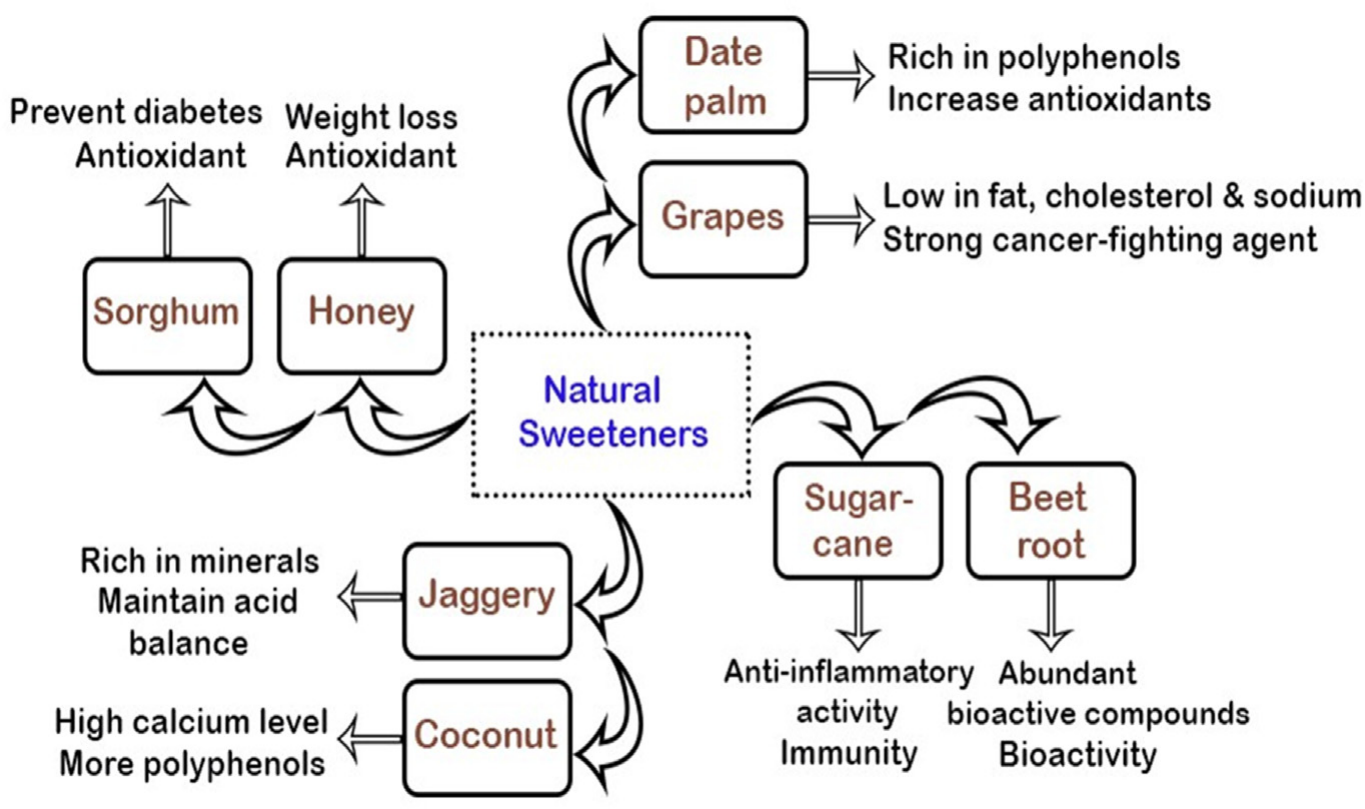
Benefits of natural sweeteners over refined sugar.

The public's growing interest in health and wellbeing has raised demand for low-calorie goods (Manickvasagan et al., 2017). That’s why many inventions have focused on the replacement of refined sugar (table sugar) with sugars from natural sources that can be used in solid, semi-solid and liquid food applications. Sugars from natural sources (unrefined sugar) include a variety of bioactive compounds, minerals, fibers, antioxidants, and phytochemicals that help to decrease inflammation as well as enhance endothelial function. The nutritional properties and antioxidant potential of sugars are influenced by the degree of purification. The research found that unrefined sugars might have a positive dietary impact if they are substituted for refined sugars, due to higher phenol and flavonoid content (Lee et al., 2018). Many studies have highlighted the potential benefits of antioxidant activity in unrefined sugars. For example, research in adults suggested that weight gain is inversely related to naturally occurring sugars (Fidler Mis et al., 2017). Other benefits of natural sweeteners are shown in Figure 2.

Consumers are becoming more worried about their health, which is leading to a rise in interest in eating healthier meals. In this context, there has been a need for more natural, and healthier sugars to replace typical white sugar (Curi et al., 2017). Therefore, sugar from natural sources is chosen because they have proven more health benefits compared to refined sugars. Many plant-based sources can be considered alternative sources of sugar. In addition, all plant-based sources also provide additional nutritional value and have the potential to protect against certain diseases instead of providing just empty calories. Some prominent natural sweeteners that can be used as substitutes for refined sugar are as follows:

### 3.1 Date palm

Date fruit has many important bioactive components. In Africa and Middle East, dry and fresh dates are used and grown since the ancient era. All over the world, there are about 5000+ date palm varieties which differ from each other based on nutritional composition, genetic factors, and morphological factors. *Phoenix dactylifera* generally called date palm is considered the most important socioeconomic tree among all varieties since it can be used as raw materials for a wide range of foods. Dates are an excellent source of carbohydrates, the major ones being glucose and fructose (Shehzad et al., 2021).

In 2011, the production of dates in Pakistan ranked seventh amongst all other date-producing countries. 150+ varieties of dates are cultivated in Pakistan, which includes Begum Jangi, Dhakki, Aseel, Halavi. These are commonly grown in Sindh, Balochistan and some districts of Punjab. Dominant varieties are Dhakki and Aseel (Nadeem et al., 2019). For producing palm sugar, a huge amount of sap is filtered and transferred to a vessel where it is heated at around 100 °C by a wood fire for a few hours. It is taken off when it becomes thick and concentrated. After heating, the liquid from palm sap is poured into bamboo moulds to form a pure solid palm ready for use in sugar (Srikaeo and Thongta, 2015).

Dates are emerging as a replacement for refined sugar. There is great possibility and potential to produce date fruit syrup for usage as a replacement for sucrose in food products. Research has shown the strong contribution of dates to human health as depicted in Table 2. Based on dry weight, dates have 10–20% moisture, 60–75% total sugars, nearly 2% protein, 5–8% fiber and less than 1% fat (El-Sharnouby et al., 2014).

**Table 2:** Reported benefits of the date palm and its mechanisms.

Reported Benefits	Mechanism and detail	References
Fertility Enhancement	Results of a rat study show that during copulation, dopamine releases and facilitates genital reflexes, sexual motivation, and motor performance. Dopamine has been known to aid sexual function in males. The presence of certain substances in date palms like α-amirin, estrone, estradiol, estriol, triterpenoidal saponins, and flavonoids explains the aiding part of the fruit on sexual functions by elevating the discharge of dopamine in the hypothalamus.	(Abedi et al., 2014)
Beneficial phytochemical content	The date fruit is rich in active compounds like phytochemicals such as tannins, carotenoids, polyphenols (e.g., phenolic acids, isoflavones, lignans, and flavonoids), and sterols.	(Martín-Sánchez et al., 2014)
Protection against diseases	Studies conducted on extracts of date palm either pure aqueous or mixed with organic solvent found that it has many important constituents that have health benefits like oxidative stress activity, prevention of coronary heart disease and cancer, free radical scavenging activity, liver protection and anti-inflammatory activities.	(Al-Alawi et al., 2017)
Against Alzheimer’s Disease	A rat study suggests that supplementing the diet with date fruits may help with delaying the onset of Alzheimer’s Disease as well as reducing the risk of slowing down the progress of this disease.	(Subash et al., 2015)
Gut health	Data suggests that consuming date fruits can modify colon health by preventing the spread of cancer cells in the colon and by increasing the growth of beneficial bacteria.	(Guardiola et al., 2016)
	The laxative property of *P. dactylifera* fruit has been verified by checking its effect on gastrointestinal transit time in mice. Results showed that the mice that received date fruit extract emptied more of their GIT content due to high fibers.	(Sani et al., 2015)
Source of minerals	Consuming 100 g of dates bring about 15% of recommended daily allowance of various important minerals, including copper, iron, calcium, magnesium and potassium. Dates also contain generous amounts of vitamin B2 and B3 and total phenolic contents	(Nadeem et al., 2019)
Anti-hyperlipidemia and hepatoprotective activity	A study conducted on hyperlipidemia-induced albino rats shows positive results in preventing hyperlipidemia and fatty changes in the liver of rats. This consider able antihyperlipidemic activity may be due to flavonoids and polyphenols' presence in dates.	(Ahmed et al., 2016)
Defense against oxidative damage	Date fruit has great reducing power, free radical scavenging activity and antioxidant activity the antioxidant potential was attributed to phytoconstituents (flavonoids, saponins, tannins, steroids) and vitamin C.	(Sani et al., 2015)
Liver protection	A study was done on rats where they were fed with aqueous extracts of *P. dactylifera*. Results showed a reduction of carbon tetrachloride (CCl4) induced a rise in plasma enzyme and bilirubin concentration and reduced liver damage.	(Sani et al., 2015)
Anticancer or anti-tumor properties	Invitro's study shows that the presence of phenolic cancer prohibition factors (such as flavonoids, sinapic acid, ferulic scan and procyanidins) explains this property. These phenolic compounds have antioxidant properties and act as cancer chemopreventive compounds, thus obstructing carcinogenesis at the initial stages.	(Hamed et al., 2017)
Protection against kidney damage	Results of a study where rats were fed with extract of *P. dactylifera* fruit. Decreased levels of plasma concentrations of creatinine and urea were observed. And it could reduce the gentamicin-induced damage to the proximal tubular areas of the rat kidneys.	(Sani et al., 2015)

Fructose from date plant reduces glycaemia after eating, as it gets discharged into the blood in a small amount to reasonable concentration in healthy as well as hyperglycemic individuals (Younas et al., 2020). Date fruit and its seed have medicinal and nutritional value. The date is rich in phytochemicals such as phenolic compounds. Experimental trials using dates have indicated effective results against many cancers (Maqsood et al., 2020).

### 3.2 Grapes

Grapes are an economically significant fruit crop worldwide. World grape production is about 22.15 million metric tons during the year 2018/2019. A large amount of the grape harvest is used for winemaking and also eaten as fresh fruit while some grapes are used to make raisins (Lee and Sumner, 2015). Fresh grape juice usually consists of water (70–80%) and many dissolved solids that are composed of many organic and inorganic compounds. For winemaking, the important groups of compounds are sugars, organic acids, phenolic compounds, minerals and pectic substances (Tarko et al., 2014).

A large number of soluble solids in grapes are sugars, especially glucose and fructose. The sugar content ranges from 150 to 250 g/L of ripe grapes. The main sugar in unripe grapes is glucose. Fructose and glucose are in equal amount at the ripening stage (1:1 ratio) (Jones and Goodrich, 2008), while the concentration of fructose in overripe grapes is above that of glucose. There is some difference between the grape varieties in the ratio fructose and glucose in ripe grapes. The sweetness of fructose, glucose and sucrose varies considerably, with fructose being the sweetest. Therefore, less fructose than sucrose is needed to achieve the same sweetness (Shiraishi et al., 2010).

### 3.3 Sugar beet

The sugar beet plant contains a higher concentration of sucrose in its roots. It is grown commercially for the manufacturing of sugar. The sugar beet has a flat crown and conical, thin, fleshy roots. The plant is made of a root and a leaf rosette. Sugar is produced in the leaves through a photosynthesis cycle and then accumulates in the roots. The beetroot contains 75% water, about 20% sugars (between 12 and 21% depending on the cultivar) and 5% pulp (Ahmad et al., 2012). The pulp involves mainly cellulose, hemicellulose, lignin, and pectin. It is water-insoluble and can be used in animal feed. Sugar beet crop by-products, such as pulp and molasses, account for another 10% of the harvest value (Habeeb et al., 2017).

The pulp is a waste product in sugar-beet processing after sugar extraction. Favorable tactile, physical, chemical, sensory and microbiological characteristics mark pulp as an important source of dietary fibers (Thalooth et al., 2019). Compared to cereal bran, low phytate is one of the distinguished properties of sugar beet fiber, which is a very important aspect for nutritionists since phytates are known to impair mineral absorption. It has good water holding and retention ability, which is a very good characteristic of the baking industry (Habeeb et al., 2017).

### 3.4 Sweet sorghum

Sweet sorghum has been labeled as a favorable crop with the capacity to provide a wide range of energy uses. It contains low sulfur content, a high caloric amount and a near-zero carbon dioxide balance, making it a non-polluting energy source. The chemical composition of sweet sorghum juice includes sucrose, glucose and fructose, xylose, ribose, arabinose, sorbose, galactose, mannose, and poly glucose (Abdelmuti and Taiseer Hassan, 2019).

As a valuable source of glucide (several organic compounds that comprise carbohydrates), sweet sorghum is a potential source of juice and a variety of quality foods. Sorghum can be used as a subs for refined sugar, sorghum juice contains macro and microelements (especially potassium, calcium, and manganese) and amino acids, which make sorghum an important nutritional product (Abdelmuti and Taiseer Hassan, 2019).

Sorghum has certain health benefits for consumers because of the quality of its carbohydrates, and the presence of other chemicals that are typically considered anti-nutrients in animal nutrition, such as polyphenols or tannins and polycosanols (Awika, 2017).

Tannins can increase food intake and digestive enzyme production to reduce weight gain while polycosanols are known to increase lipoprotein concentrations and boost cardiac function. Sorghum is also gluten-free and also beneficial for celiac sufferers (Mabelebele and Iji, 2013). The syrup, obtained from sweet sorghum, is like natural honey in its composition of biologically active constituents and microelements. Data on the carbohydrate content of sorghum syrup institute the prospect of its use in bakeries and pastries. For baking, sugar can be substituted from 10 to 100% and in confectionery, from 10% for marmalade, 6% for jellies and 15% for caramel filling. Sorghum syrup can be used to substitute sugar, partially or completely within the fruit compote and jam production networks.

### 3.5 Sugarcane

Sugarcane is the main source (90%) produce sweeteners. Sugar cane can grow up to 4.5 m high. It is related to the genus Saccharum and is a member of the grass family having six species: namely, *S. officinarum*, *S. Barberi*, *S. sinense*, *S. robustum*, *S. sponteneumand*, and *S. elude* (Kocher et al., 2014). Agro-processing industries use sugarcane as an important raw material to produce a variety of sugars, jaggery, and Khansari. India contributes to producing the largest amount of sugar in the world and produces about 70% of jaggery in the world (Said and Pradhan, 2013). Sugarcane (about 37.2%) was used to produce jaggery and Khandsari (Nath et al., 2015).

Sugarcane was long divulged and cultivated as an essential sugar and biofuel source. Sugarcane is mostly valued in Europe and North America for its sweet taste; however, sugarcane was utilized as medicine in the subtropics and tropics where it is cultivated. In Ayurvedic medicine, various health complications such as influenza, constipation, cough, anemia, bronchitis, jaundice, general weakness and blood disorders are cured by sugarcane (Edwards et al., 2016). The medical importance of sugarcane is less known in the world, although traditional Indian medicinal systems have long recognized sugarcane as a cure for septic shock and various other illnesses (Ji et al., 2019).

Current pharmacological studies have found several phytochemicals in sugarcane, including polyphenolic compounds such as phenolic acids and juices, and various glycosides and their other products (e.g., molasses). In recent years, sugarcane has raised interest in nutraceutical products. Many studies have revealed valuable effects of sugarcane extracts in vivo models in stimulating the immune system, protecting against liver injury, recovering intestinal working, protecting various infections, anti-stress outcomes and stimulating development (Edwards et al., 2016).

In addition, sugarcane possesses strong antioxidant functions and plays a vital role in health since it is believed that many human diseases such as cancer, cardiovascular illnesses or other degenerative ailments are caused by oxidative damage (Seguí et al., 2015). Sugarcane’s antioxidant properties are generally due to phenolic compounds, especially flavonoids, phenolic acids, and polyphenols. It is thought that phenolic food compounds, particularly flavonoids could have favorable effects on human health. Such compounds, however, are undesirable components in the making process of sugar and are detached from juice through processing (Arif et al., 2019; Khan et al., 2021; Zia et al., 2019).

Certain products manufactured with sugarcane, such as molasses, may possess antioxidant properties because of Maillard reactions, which occur during manufacturing. Several researchers have revealed antioxidant properties in both raw sugarcane and molasses (Singh et al., 2015). Among other advantageous properties, antiradical ability, lipid peroxidation inhibition, and defense against oxidative and antiproliferative activity against cancer cell lines were recorded (Abbas et al., 2014).

### 3.6 Jaggery

Jaggery (or Gur) is a natural sweetener acquired through concentrating the sugarcane, and sweet juices in a solid or semi-solid state with or without previous juice purification and the use of any synthetic additives, chemicals, or preservatives. While known as the sugar of the poor man, Gur is consumed in the subcontinent (Gartaula and Bhattarai, 2014). Jaggery is deliberated as a food ingredient, since it is comprised of a large number of minerals and vitamins along with energy and is consumed as a sweetener directly, as well as in various preparations like mixtures of animal feed. For instance, farmers feed jaggery cake to pigs during winter in the sugarcane belt. The jaggery filter cake is a good source of exceptional energy and minerals. Jaggery is one of mankind's oldest sweeteners and is an important part of the diet in many countries like India, especially in rural areas.

There are three types of jaggery, which are solid jaggery, liquid jaggery and granular jaggery (Said and Pradhan, 2013; Singh et al., 2013). Jaggery’s micronutrients have numerous nutritional and therapeutic effects, such as their anti-carcinogenic and antitoxic activity. Jaggery is a sugarcane product, which is significantly rich in minerals and vitamins. The major vitamins found in jaggery are vitamin A (3.8 mg), vitamin C (7 mg), and vitamin D2 (6.5 mg). Other important minerals in jaggery are Calcium (40–100 mg), Magnesium (70–90 mg), Potassium (10–56 mg), Phosphorus (20–90 mg), Sodium (19–30 mg), Manganese (0.2–0.5 mg), Zinc (0.2–0.4 mg), Copper (0.1–0.9 mg), Chloride (5.3 mg), and Iron (10–13 mg) (Singh et al., 2013).

As compared to sugar, jaggery has proven to be more effective owing to its instant energy production and prevention of insulin resistance, abdominal adiposity and other chronic diseases (Mahalaxmi and Hemalatha, 2018). Jaggery is commonly used in the production of sweet dishes in India and is a widespread part of the Indian subcontinent's cuisines in the processing of many sweet dishes such as candy, toffees, jaggery cakes and other similar sweet dishes such as payasam, obbattu (holige) and unday (Jaffé, 2012).

The granular jaggery contains 0.6–1% minerals such as 9 mg pure calcium, 4 mg phosphorous and 12 mg iron. Jaggery is more composite than sugar as it consists of some amount of mineral salts, and a longer chain of sucrose, iron and fiber. Compared to sugar, jaggery powder has a significantly higher moisture content, ash content, lower total sugar, and lower protein and fat. The overall quality score for jaggery muffins was lower than that for sugar muffins. It can be concluded that in the future, jaggery can be utilized in different baking products on an equal weight basis of sugar (Lamdande et al., 2018).

Bomboyson is a conventional dairy product made from Khoya with ghee and sugar. Bomboyson is a sweet candy, colored dark brown with a slightly caramelized taste and produced in a rectangle shape in Nepal. Few local manufacturer’s dairy industries produce low-scale bomboyson. Sugar which is used as a sweetener in bomboyson is devoid of vitamins, minerals, and phytochemicals. Jaggery compared to sugar is wholesome and contains 65–85% sucrose compared with 99.5% sucrose present in sugar (Gartaula and Bhattarai, 2014).

### 3.7 Honey

Honey is a naturally occurring sweetener that has a complex composition. It consists of about 200 components, mainly comprising sugars (75% are monosaccharides, 10–15% are disaccharides with traces of other sugars). Moreover, there are other components such as water, enzymes, proteins, phenols, vitamins (especially B vitamins), minerals, organic acids, and solid particles from harvesting. More than 38% of fructose and about 30% of glucose are found in honey. High sucrose levels may show that there might be addition of some adulterants and cheap sweeteners, or early harvest indicating that sucrose did not get completely converted into fructose and glucose (da Silva et al., 2016).

For a long time honey has been used for medicinal purposes. Apitherapy is a branch of medicine that offers honey-based treatments and other bee products against many diseases. Studies have shown honey can be a preventive agent against oxidative stress disorders including cardiovascular disease, cancer, diabetes, hepatic and renal failure and aging. There are three different types of honey based on color shades which can be light, amber, and dark. Oligosaccharides present in honey might benefit gut health by encouraging the growth of beneficial bacteria (*Streptococcus thermophilus*, *Lactobacillus acidophilus*, *Lactobacillus* or *Bifidobacterium bifidum*) in the colon (Ismail, 2017). The color, fragrance, composition and flavor of honey are chiefly dependent upon the flowers, geographic regions, processing, handling, packing, storage time, climate and species of honeybee involved in its production (Escuredo et al., 2014).

Results of many studies have shown honey to be a possible anti-obesity agent that helps to control obesity and overweight. Data from human studies showed that honey reduced their body weight, body fat and also cholesterol. Another study on overweight subjects showed that consuming honey reduced triglycerides and LDL: low-density lipoprotein cholesterol and slightly increased HDL high-density lipoprotein cholesterol (Samat et al., 2017). Table 3 shows some beneficial effects of natural sweeteners.

**Table 3:** Reported studies on the compounds and beneficial effects of natural sweeteners.

Natural sweeteners	Compounds	Reported Health Benefits Inhibiting Toxicity and Adverse Reaction Issues	References
Date palm	Dietary fiber, minerals, carotenoids, vitamins and polyphenols	Cell biology techniques were used in a study to demonstrate date palm polyphenols that inhibited hIAPP and prevented T2DM Reduced cytotoxicity and aggregation	(Chaari et al., 2020)
Grapes	Flavonoids, fructose, pectin, proanthocyanidin	A study on rats reveals that grapes inhibited myocardial infarction and prevented lung, breast and gastric adenocarcinoma cells Combat the free radicals to avoid oxidative stress	(Bagchi et al., 2000)
Honey	Niacin, water, riboflavin, potassium, copper, trace amounts of vitamins	MSA mice were fed with 10% honey and the result demonstrated the prevention of mycotoxins genotoxicity for two months Enhanced the gut microflora to provide antigenotoxicity and detoxification	(Ezz El-Arab, Girgis, Hegazy, El-Khalek and Azzat, 2006)
Sorghum	Bioactive compounds, polyphenols	Antioxidant activity prevents the risk of several cancers, decreases the availability of calories and reduces weight gain, hence preventing obesity, cardiovascular diseases and cancer effectively	(Awika and Rooney, 2004)
Sugar-cane	Vitamin A, Vitamin B Complex, minerals	A research study on FW rats revealed OARC and antioxidant properties Inhibited peroxidation of lipid, scavenge free radicals and prevent iron complex	(Kadam et al., 2008)
Beetroot	Betalains, bioactive compounds,	In vivo and in vitro trials showed anti-inflammatory, anti-oxidative and chemo-preventive action	(Clifford et al., 2015)
Jaggery	Vitamins, Magnesium, calcium, zinc	MSA mice were fed with jaggery (250mg/mice) to determine the antioxidant effect for 180 days Prevented the DNA damage, showed degenerative and necrosis alternations in bronchiolar epithelium	(N. Singh, Kumar, Lal, Raisuddin and Sahu, 2010)
Coconut	Iron, calcium, zinc and potassium, polyphenols and antioxidant	Prevent gum disease, diabetes development and heart disease and several other serious health issues	(Trinidad, 2003)

hIAPP = human Islet Amyloid Polypeptide, T2DM = Type-2 diabetes mellitus, MSA = Male Swiss albino, FW = Female Wistar, OARC = oxygen radical absorbance capacity.

### 3.8 Non-nutritive sweeteners (NNS)

NNS are often used to substitute refined sugar (Wilk et al., 2022). These sweeteners can be natural or artificially produced substances that have a higher sweetening capacity than sugar, and no caloric content (Silva Monteiro et al., 2018). The European Food Safety Authority (EFSA) has also suggests that within limited amounts these substances are safe for human use (Wilk et al., 2022). In the United States, The Food and Drug Administration (FDA) has approved eight artificial sweeteners including two natural sweeteners (stevia and monk fruit extract) and six synthetically produced sweeteners (aspartame, acesulfame potassium (Ace-K), neotame, saccharin, sucralose, and advantage) (Basson et al., 2021).

Artificial sweeteners cannot simply substitute natural sugars in terms of metabolism because humans can readily metabolize them and thus have a low-calorie content since they are not entirely metabolized in the human body (Ali et al., 2021). Natural sweeteners have far superior physical properties to artificial sweeteners in terms of sweetness intensity, quality, degradability, and quantity in nature (Ali et al., 2021). Natural NNSs, such as stevia and monk fruit, are produced from plants and include natural components, therefore they may have a higher level of consumer acceptability (Mahato et al., 2021).

#### 3.8.1 Monk fruit

Monk fruit (*Siraitia grosvenorii*), also known as Luo Han Guo, is a perennial plant that is extensively grown in China's Guangxi province (Pandey and Chauhan, 2019). Monk fruit possesses anti-cancer, antioxidant, anti-inflammatory, anti-obesity, and antidiabetic properties, among other health benefits (Mahato et al., 2021). In a recent study, it was concluded that extract of monk fruit showed a noteworthy drop in insulin resistance (Ban et al., 2020). Monk fruit is also a low-cost, high-intensity natural sweetener that may be used in the food and beverage sectors to create low-calorie goods for subjects with diabetes and health-conscious customers (Mahato et al., 2021).

#### 3.8.2 Stevia

*Stevia rebaudiana* Bertoni (stevia) is a South American herb associated with the sunflower family (Asteraceae). Scientific data shows that stevia leaves comprise essential amino acids, carbohydrates, protein, phenols and antioxidants like ascorbic acid. Thus, stevia leaves or extracts might be considered not only natural sweeteners but can also be used as means of food preservation (Bender et al., 2015). The compounds that are accountable for the sweet taste are stevia glycosides (e,g, stevioside is around 300 times sweeter compared to saccharose). These are low-calorie and non-toxic sweeteners, which makes them a good substitute for sucrose in food and beverages. Many studies suggest that it can be particularly helpful for diabetes, dental carry, heart diseases, hypertension, obesity and dental carries prevention. Out of more than 200 species in the genus *Stevia*, only the species rebaudiana and phlebophylla produce steviol glycosides (Lemus-Mondaca et al., 2012).

An analysis of dried leaves of stevia shows its low energy value of 2.7 kcal/g. *Stevia* plants contain essential amino acids, fatty acids, some vitamins, minerals, prebiotic and anti-inflammatory properties and also dietary fiber which helps in better control of blood glucose level (Putnik et al., 2020). From numerous in vivo and in vitro animal studies and also some human tolerance studies on the steviol glycosides rebaudioside A and stevioside and their metabolite steviol, it was concluded that these compounds are non-carcinogenic, non-genotoxic and not connected to any toxicity. Therefore, it can be said that these are safe to consume (Additives and Food, 2010).

### 3.9 Physico-chemical properties of natural sugars

Natural sweeteners exhibit physicochemical properties concerning their usage in different food products. Total soluble solids, color and pH of natural sugars play a key role in determining their potential use. The total soluble solids (°Brix) values are very similar among all-natural sweeteners ranging from 65 to 79.5 (Mellado-Mojica and López, 2015). Along with other physicochemical properties such as high osmolarity and viscosity, the acidic nature of honey (with a pH in the range of 4–5) has an impact on its health effects (Truzzi et al., 2014). In the course of rheological studies, thermal stress was applied to creamed honey which revealed thermal stability and exhibited great structural alternation between 5 and 50 °C. Hence, the study suggested that the thermal fluctuations should be avoided to maintain crystallized honey and should be kept at below 40 °C to prevent irreversible alternations and, to persist spreadability (Karasu et al., 2015). Natural honey is sparingly soluble at room temperature while powder honey extracted by spray drying is soluble in water completely and the solubility time of honey ranges from 28 to 148 s. Natural sugars such as sugarcane, beetroot, date palm, jaggery and grapes show thermal stability and solubility through processing and production (Samborska and Bienkowska, 2013).

## 4 Artificial sweeteners

Artificial sweeteners are synthetically produced substitutes for sugar, whose sweetening power of sucrose is significantly higher per unit of weight (up to 4000 times sweeter, depending on the type) (Dhartiben and Aparnathi, 2017). They have very few or no calories and are not carbohydrates (Saraiva et al., 2020). Acesulfame K, advantame, aspartame, cyclamate, neotame, saccharin, and sucralose, are to be distinguished from sugar substitutes such as sorbitol, maltitol or xylitol, which have a much lower sweetening power and are modified sugars (sugar alcohols) (Carocho et al., 2017). In practice, artificial sweeteners are usually mixed with sugar substitutes to make the sweetness of the products more pleasant and in particular to mask the often slightly bitter aftertaste of some artificial sweeteners (Wilk et al., 2022).

Artificial sweeteners are controversial, and the health authorities of different countries have different opinions. Some are banned in the United States due to suspected cancer risks, however, are permitted in the European Union (Landrigan and Straif, 2021). Several studies have shown that artificial sweeteners raise insulin levels because the body responds to sugar when the signal “sweet” is received. If the “sweet” signal comes from an artificial sweetener rather than sugar, the blood sugar level drops as a result of the insulin secreted by the body as a precaution, resulting in hunger attacks (Bueno-Hernández et al., 2020).

This effect is controversial among experts, and there have been studies that have been unable to confirm it. Many consumers prefer to reach for artificially sweetened foods - either hoping to lose weight thanks to the lower-calorie sugar substitutes or considering these sweeteners to be less harmful to health than sugar. Unfortunately, there is no clear evidence so far that artificial sweeteners are effective in helping people lose weight (Pang et al. 2021; Wilk et al., 2022). On the contrary, recent studies suggested that the long-term use of artificial sweeteners is more likely to lead to weight gain and associated health risks such as high blood pressure or type 2 diabetes (Lohner et al. 2017; Higgins and Mattes, 2019). Some of the most commonly used artificial sweeteners - saccharin, aspartame and sucralose – can cause health problems by affecting the bacterial layer in the gut, called intestinal flora, and can cause for example two types of intestinal bacteria – *Escherichia coli* and *Enterococcus faecalis* – to behave in ways that are harmful to health (Lobach et al., 2019; Ruiz-Ojeda et al., 2019). The pathogenic changes in the intestinal flora are, on one hand, a stronger formation of biofilms and, on the other hand, increased adhesion and invasion of bacteria into human intestinal cells. Biofilms are layers of mucus formed by microorganisms that are themselves embedded in this mucus layer. Since these biofilms protect the bacteria embedded in them from the immune system and make them less sensitive to antibiotics, they pose a health problem (Deng et al., 2020; Domingue et al., 2020).

Sucralose and aspartame also caused the intestinal bacteria to attach to certain cells that line the intestinal wall. The microorganisms then infiltrated these cells and killed them. In this way, bacteria such as *Enterococcus faecalis* penetrate the lymph nodes and accumulate in the liver and spleen, which can lead to infections and even sepsis (colloquially “blood poisoning”) (Basson et al., 2021; Shil and Chichger, 2021). The scientists emphasize that there is still a lot not understood about artificial sweeteners, which are added to many foods, and further research is urgently needed (Serrano et al., 2021; Wilk et al., 2022).

## 5 Emerging technologies

Sugar reduction and replacement are currently one of the highest priorities in product innovation. Consumer demand for a better lifestyle via more natural, nutritional products. Particularly obesity has been identified as a severe illness from COVID-19. Currently, new bioprocesses as emerging solutions challenges are proposed such as to increase batch output volume (e.g. xylitol) (Queiroz et al., 2022; Vollmer et al., 2022) or to produce sweeteners from renewable and sustainable sources than conventional ones. Good examples are represented by steviol glycoside sweetener that could be produced via chemical and enzymatic reactions e.g. fermenting bio-wastes (e.g. sugarcane) with yeasts (or tagatose, a natural sugar found naturally in some fruits (apples, pineapples, and oranges) and in smaller amounts in processed dairy products - heated cow's milk and fermented products such as yoghurt that be produced from agricultural waste resources cornstarch. Researchers and companies have committed themselves to continue working on sugar reduction and replacement, but at the same time, consumers must also buy the improved products. The efforts are put not only into optimizing recipes, but also into creating new products, which is difficult to do without sweeteners (McCain et al., 2018).

## 6 Conclusions

Using refined sugar in our daily life is a common practice, and its usage has increased many folds over the last decades. It is used commonly in desserts, tea, milk, juices, shakes and bakery products in every home all over the world. However, it is very threatening to human health. Many chemical additives are used in the discolouration and other extraction and preparation processes, which can be harmful. Evidence has shown that sugar causes many health problems including obesity, metabolic syndrome, diabetes, dental caries, elevated cholesterol, high blood pressure and might even cause cancer. It could also contribute to impaired glucose tolerance and insulin resistance, which have been associated with cognitive impairment, thus affecting the brain harmfully. In addition, it can cause complications in pregnancy. Therefore, various approaches have been proposed to avoid these health problems by reducing the usage of sugar and thus replacing it with better nutritional alternatives. The best ones in this regard are naturally occurring sweeteners such as honey, date palm, jaggery, sugar beet, sugarcane, sorghum, and grape sugar. In addition to their high content of natural sugars, these natural products also contain many vitamins, minerals, phytochemicals, antioxidants, and other healthy substances. These natural products help in maintaining overall gut health and well-being of the body in addition to acting as sweeteners. Some of these natural substances have also been used for medicinal purposes for a longer time. These alternative sugars used as sweeteners do not pose any harm to human health as sugar does, and instead, they provide additional health benefits.

Due to the modern lifestyle and changes in consumer eating habits, sugar intake has increased massively in recent years. Future studies should investigate better and more applicable ways to use these natural products as sweeteners in daily life to replace refined sugars. The problem might be that these food items are mostly not cheap, and many people find them difficult to afford. Cheap, easily available and handy forms of these products must be introduced to the markets so that consumers can easily benefit from them. Governments should take a step in this regard by organizing public information campaigns and seminars, especially in less privileged areas to educate consumers about the health dangers of consuming excess refined sugars while promoting the positive aspects of sugars from natural sources.

## Declarations

### Author contribution statement

All authors listed have significantly contributed to the development and the writing of this article.

### Funding statement

This research did not receive any specific grant from funding agencies in the public, commercial, or not-for-profit sectors.

### Data availability statement

Data included in article/supp. material/referenced in article.

### Declaration of interest’s statement

The authors declare no conflict of interest.

### Additional information

No additional information is available for this paper.
